# Beyond depressive symptoms: conceptualizing social suffering among displaced Syrians in Lebanon

**DOI:** 10.3389/fpubh.2026.1844218

**Published:** 2026-06-19

**Authors:** Laura Hertner, Dana Abdel-Fatah, Emily Frank, Ulrike Kluge

**Affiliations:** 1 Berlin Institute for Empirical Integration and Migration Research at the Humboldt Universität zu Berlin, Berlin, Germany; 2 Department of Psychiatry and Neurosciences at the Charité Campus Mitte, Charité – Universitätsmedizin Berlin, Corporate Member of the Freie Universität Berlin and Humboldt-Universität zu Berlin, Berlin, Germany; 3 Einstein Center Population Diversity (ECPD), Berlin, Germany; 4 Deutsches Zentrum für Psychische Gesundheit (DZPG), Standort Berlin-Potsdam, Berlin, Germany; 5 WZB Berlin Social Science Center, Berlin, Germany

**Keywords:** armed conflict, feeling broken or destroyed, Lebanon, mental burden, PHQ, refugees, Syrians, social suffering

## Abstract

**Background:**

Mental burden is largely conceptualized through mental illness, yet it can manifest beyond diagnostic categories. We contend that *social suffering* requires separate consideration, as it frames mental burden through intersubjective, collective, and power dynamics, while centering its inextricable link to social and political injustice. Lebanon offers a relevant setting, hosting Syrian refugees amid economic crisis and intermittent armed conflict.

**Objective:**

We assess social suffering with the novel feeling broken or destroyed scale (FBD) and compare it with a standard mental health screening instrument for depressive symptoms, namely the patient health questionnaire (PHQ). We contend that both these complementary notions of social suffering and depressive symptoms measure separate, though related, aspects of mental burden and warrant attention.

**Methods:**

Using data from the TRANSMIT survey collected among Syrians (*N* = 1,228) in Lebanon (Oct–Dec 2023), we conducted a regression analysis comparing the predictors FBD and PHQ and explored each construct's sensitivity for the broader socio-political context. Conceptually overlapping items (hope for Syria/Lebanon's future) were excluded from the regression and tested via one-way ANOVA.

**Results:**

Though used for the first time within the Lebanese context and among a displaced sample, the FBD showed good internal consistency (Cronbach's Alpha = 0.822; Omega = 0.838). PHQ and FBD demonstrated moderate positive correlations in our sample (ρ = 0.56, all *p* < 0.001), indicating that they are sufficiently distinct. Regression analysis indicates that the FBD may be more sensitive to some socio-political factors, such as poverty, poor neighborhood quality, and experiences of discrimination, reflecting more enduring forms of mental burden, including apathy and exhaustion. In contrast, harsh working conditions characteristic of day laborers as well as residence in an area of active armed conflict may foreground acute, somatic and discrete symptoms, such as sleep disturbance or appetite changes, detected by PHQ.

**Conclusion:**

Reframing mental burden among displaced Syrians in Lebanon through the lens of social suffering rather than mental illness can help capture the effects of structural vulnerabilization. This encompasses the cumulative impact of material deprivation, social exclusion, and marginalization, while pointing to resistance as a response to these conditions.

## 1 Introduction

Lebanon has one of the world's highest refugee-to-population ratios, with more than 1.5 million Syrians having fled the civil war across its borders (1). The mental health of refugee populations has received sustained international attention, as both the violence preceding flight and the context of displacement, marked by precarity and loss, constitute significant and compounding stressors (2–9). The Lebanese context warrants particular attention in this regard, as refugees arrive in a country that is itself in profound crisis: an economic collapse, political paralysis, recurrent armed conflict and a state infrastructure increasingly unable to meet the basic needs of its own population. Against this backdrop, we propose that a comprehensive assessment of the mental burdens of the displaced Syrian population in Lebanon requires a notion of suffering that extends beyond clinical definitions of mental illness. We present our attempt to capture social suffering with the Feeling Broken or Destroyed scale (FBD) in a structured survey and compare it with a standard mental health screening instrument for depressive symptoms, namely the Patient Health Questionnaire (PHQ). Through a regression analysis, we explore the predictors of these two distinct constructs and their sensitivity to the broader socio-political context of the community. We suggest that both these complementary notions of social suffering and depressive symptoms warrant scholarly attention.

### 1.1 Theoretical background: psychopathology and social suffering

The biomedical paradigm conceptualizes mental burdens as falling within psychiatry's domain, equating them with mental illnesses (10, 11). In contrast to subjective reports of mental burden or suffering, mental illnesses diagnosed based on standardized DSM (the American Psychiatric Association's Diagnostic and Statistical Manual of Mental Disorders) or ICD (the World Health Organization's International Classification of Diseases) criteria are regarded as *objective* measures of health. As such, mental illnesses offer reliable recognition of mental burden, access to medical and psychological treatments, and opportunities for compensation, pension and other legal claims. For refugees, mental illness, may under certain conditions support appeals against deportation–sometimes even discussed a prerequisite for the right to stay (12–14). While mental burdens are largely associated with diagnosed mental illness and severely impaired mental and behavioral functioning, our research and clinical experience suggests that significant mental burden exits beyond these diagnostic categories. Although this burden forms part of the real experiences of suffering, it is rarely considered in public health research. The dominant focus on mental illness thus risks obscuring mental burdens that do not qualifying for diagnostic categories such as depression, anxiety, or post-traumatic stress disorders (PTSD)—categories particularly prevalent among studies on refugees' psychopathology.

A further limitation of focusing on mental illness is that it may obscure root causes and context-specific meanings through individualization, pathologization and de-politicization of the mental burden (15–20). Diagnostic categories reduce the effect of experiences of structural violence, for instance, to a “catalog of standardized” symptoms at the individual level [(21), p.35]. Consequently, when mental and behavioral impairment is framed as pathological, *individuals* are labeled as “ill” and seen as in need of help, medication, or therapy (17, 22). While psychopathology spotlights mainly the intrapsychic processes, it is necessary to equally consider the intersubjective, collective and power dynamics in and from which the mental burden arises and their inextricable link to (transgenerational) and at times chronic social and political injustice (17, 23–25). This perspective is particularly relevant within communities where mental burdens represent shared experiences tied to socio-political contexts. Such communities include those displaced by war and violence, where de-politicization is particularily problematic.

The concept of *suffering* offers a broader analytical lens “to articulate the unbearable” [(18), p. 57]^1^, without initially differentiating between normal and abnormal, as psycho*pathology* does. By tying suffering to the “social context where [the lived experience of individuals] are formed” [(18), p. 56], and “describing the social process of suffering, [it] helps us to stop assigning responsibility to [...] individuals and contributes to a co-construction of suffering” [(18), p. 10]. Thus, *social suffering* may serve as a relevant conceptual supplement that, enables us to center the collective and socio-political aspects of suffering—as a normal reaction to abnormal circumstances of war, structural violence, social and political injustice. It thereby, “leads to a broadening of the questions of mental health by taking into account a set of subjective harms and social processes that fall outside the traditional concerns of public health.” [(18), p. 34].

Kleinman et al. in their edited volume *Social Suffering* developed theoretical foundations for studying social suffering in contexts of war, famine, torture and “political catastrophes”. The authors argue that these “extreme forms of suffering […]” are not the same as “ordinary experiences of poverty and illnesses” [(26), p. 2]. They highlight the impact of structural violence on suffering while emphasizing its cultural dimensions and the challenges of expression and communication, not least in relation to language. The authors furthermore note that the poor and powerless are not only “more likely to suffer, they are also more likely to have their suffering silenced” [(26), p. 280]. This may reflect an intentional “erasure of social experiences of suffering” which “prevents public witnessing” and denies “the collective experience of suffering” [(27), p. 16, 17].

This theoretical framing suggests that social suffering among displaced communities warrants consideration alongside individual psychopathology. The prevailing focus on mental illness—particularly on *pre*-migration traumas diagnosed as PTSD—may obscure present social suffering: the challenging reality of living as an (undocumented) immigrant in a society that marginalizes one's existence, as is the case in the context of Syrians in Lebanon. By denying these social realities, receiving societies—numerically and socially dominant on a global scale—“diffuse their own responsibility for the suffering” [(28), p. 1]. Such denial of recognition of social suffering constitutes injustice (18, 29). A focus on social suffering rather than psychopathology contributes to “a politicization of the psychological” [(18), p. 8], or, specifically in the context of armed conflict and war, the “simultaneous personalization of war and politicization of health” (19).

### 1.2 Empirical background: from structural approaches to social suffering—the challenge of operationalization

Despite the prevailing focus on psychopathology, research in the context of refugee migration has also examined the “trajectories of suffering” (18)—the context in and from which mental burdens arise. On the one hand, effects of displacement, structural violence, social and political injustice on psychopathology outcomes are modeled within the framework of social determinants of mental health. This structural approach acknowledges the influence of macro and meso-level socio-ecological determinants on the micro-level psychopathology of the individual (4, 9). Particularly for refugee populations, a growing body of literature underscores their relevance (4, 5, 7, 9), with immigration itself recognized as a relevant social determinant of health (30). On the other hand, qualitative research has advanced important insights into conceptions of suffering beyond mental illness (14, 14, 20, 20, 31–35). Although their translation into quantitative methodologies remains challenging—as “the experience of suffering, […] is not effectively conveyed by statistics or graphs” (36)—researchers have developed culturally adaptated and context-specific measurements. For the Middle East and North Africa (MENA) region, examples include the context-specific trauma scale for Palestinians (37), a contextually grounded Afghan Symptom Checklist for mental distress (38), or the South Sudan Mental Health Assessment Scale (39). While these scales are valuable, they still predominantly adhere to individual psychopathology frameworks.

We suggest that, culturally adapted mental health/illness scales should be complemented by novel instruments for structured assessments of social suffering to assess the suffering situated at the intersection of the socio-political context, the community and the individual—distinct from the individual psychopathology. Giacaman and colleagues at the Institute of Community and Public Health (ICPH) at Birzeit University have developed and applied scales specific for social suffering among Palestinians, an Arabic-speaking community historically facing social injustice, oppression, political violence, and displacement. Among other instruments, they created structured assessments of private and collective dignity and its loss [*ihaneh* and *thul*; (40)] and expanded the World Health Organization Quality of Life instrument (WHOQoL-BREF) with context-specific distress, fears and threats allowing for traceable increases of social suffering before and after the Israeli invasion of Gaza in 2008/9—phenomena invisible with the standardized WHOQoL-BREF (41). Drawing on this body of work, the present study employs the *muhattam* scale (Arabic for *feeling broken or destroyed*), developed from qualitative group interviews with Palestinians (33). These interviews centered on how participants described a person who was not doing well at all in life, eliciting accounts of “a more existential form of social suffering that, according to them, was a function of the chronic and burdensome political and economic contexts that plague them” (p. 10) (33). Retaining interviewees' own language and original terms, Barber et al. developed the FBD as a “locally-defined (i.e., emic) measure […] that differ[s] conceptually and empirically from standard Western [mental illness] measures” (p. 2), capable of capturing these existential forms of suffering inextricably tied to contexts of protracted structural violence, social and political injustice and oppression. This distinguishes it conceptually from other mental health/illness measures such as the Patient Health Questionnaire (PHQ), an internationally established self-assessment screening instrument for depression.

We adopt an exploratory approach to investigate the distribution of social suffering, assessed with the FBD, and depressive symptoms, assessed with the PHQ, among the displaced Syrian population that fled war in their country of origin and continues living under conditions of chronic conflict, social and political injustice in Lebanon. We examine the extent to which the PHQ, as a standard mental health/illness measure, may insufficiently reflect social suffering captured by the FBD in our study. We do so by comparing the predictors of these two distinct constructs and their sensitivity to the broader socio-political context of the community.

### 1.3 Local background: Lebanon and Syrian displacement

For the study of mental burden and social suffering, Lebanon represents a critically important context, shaped by the intersection of recurring armed conflict, a long history of political dysfunction and acute economic collapse. The country's modern history has been defined by cycles of violence that have left deep structural and social traces: the civil war (1975–1990), repeated Israeli military invasions and the long-term Israeli occupation of the South. In recent years, the country and its population have been shattered by one of the world's most severe economic crises, resulting in hyperinflation and currency devaluation. The consequences of this crisis permeate essential domains of everyday life, including access to food, healthcare, housing, and employment, with particularly severe implications for displaced Syrians living in the country (42). By 2021, as the COVID-19 pandemic further amplified the collapse of the national economy, 88% of Syrian households in Lebanon were living in extreme poverty (43).

Within this context, Lebanon has received one of the largest number of refugees per capita, resulting in peak periods where one in five persons living in Lebanon is a refugee. Among these refugees, 788.000 displaced Syrians were officially registered with UNHCR by 2023 (1). Although, Lebanese law does not and has never legally recognized them as refugees, Lebanon represented the nearest escape route for many Syrians after the Syrian civil war began in 2011, given the neighboring countries' long-standing social and economic ties. Most Syrians have been left without any legal status or protection. According to government officials, Lebanon was always intended to be a country of temporary transit rather than a country of destination (44). However, many Syrians displaced by the civil war have now been living in Lebanon for over a decade. The policies implemented accordingly have left Syrians isolated economically and socially, for instance through highly restrictive limitations on their access to work permits. Persistent wariness among many Lebanese toward the Syrians population seeking refuge in their country stems from Syria's military involvement in Lebanon's civil war and the subsequent occupation (1976–2005) (45). Consequently, the displaced Syrian community faces prejudicial attitudes, discriminatory practices, and in specific situations, physical aggression (46). In 2021, 57% of Syrians reported strong considerations to move to another country to live, with experiences of discrimination being the strongest predictor of such considerations (47).

Humanitarian and scholarly attention has been directed to the mental health conditions of Syrians in Lebanon. A systematic review concluded that approximately 33% qualify for PTSD, primarily due to pre-migration and war-related experiences (48). Naal et al. (49) report a prevalence of one in four Syrians exhibiting moderate to severe depression symptoms. This finding aligns with the TRANSMIT survey (2020/21), where 26% of Syrians respondents met the cut-off criteria for moderate and severe depression symptoms (9). Depression symptoms are empirically associated with advanced age, healthcare access barriers, discrimination, social exclusion and proximity to extended family (non-household) in the same urban area (9). Both studies used the PHQ.

The data presented in this study were collected between October and December 2023, a moment of regional rupture. Following the Hamas attack on October 7, 2023, and Israel's genocidal war on Gaza, the Lebanese-Israeli border re-emerged as an active front of military escalation, with Israel launching military strikes, especially in Southern Lebanon. This indicates that the data were collected in a particularly critical period in regional and global history, with not only the political deadlock, intense inflation and severe economic crises shaping the lives and suffering of the people within Lebanon, but also active conflict, violence, and military engagements. Although large-scale escalation within Lebanese territory had not yet occurred at the time of data collection, these developments unfolded within a context of recurrent conflict and wars historically embedded in the region. In particular, the 1978 Israeli invasion and the 2006 war remain embedded in the collective memory of populations in Lebanon and the broader region. These overlapping dynamics structured everyday life in Lebanon and formed the broader socio-political conditions within which experiences of mental distress and social suffering must be understood.

## 2 Material and methods

### 2.1 Sampling

Data was collected using Computer-Assisted Personal Interviews (CAPI) by Research & Consulting House (REACH) within the TRANSMIT surveys of Syrian nationals and their neighbors in Lebanon between October 5 and December 8, 2023. The survey comprehensively addressed both migration biographies and socioeconomic participation among Syrians. Sampling employed Random Walks (i.e., predetermined walking pattern to randomly select households) in randomly selected neighborhoods (Primary Sampling Units, PSU) with above-average density of Syrians (area sampling). To create a sample as representative as possible, stratified area sampling in combination with Random Walks is an established procedure for hard-to-reach populations in the absence of registry data. Households were selected based on the nationality of the household head. Within each household, selection of the survey respondent was as well randomized.

The study included persons aged 15 years and above residing in private dwellings. Residing in formal refugee camps, detention centers, and barracks was an exclusion criteria. Potential participants were contacted at their residences by Arabic-speaking staff from the implementation partners, briefed on the study's aims and scope, and invited to take part on a voluntary basis without compensation. Following agreement to participate, interviewers supplied respondents with an information sheet covering essential study details, data protection protocols, and contact options, and obtained documented informed consent. Interviews were held at a location selected by the respondent, ideally a secluded space within their own premises. This arrangement sought to establish a secure setting conducive to disclosing personal and potentially sensitive information without third-party presence. The interviews averaged 55 min. Approximately 59.4% (*N* = 737) were re-interviewed in 2023, having participated in a previous wave of the TRANSMIT surveys, while the refreshment sample (*N* = 504) was interviewed for the first time. More detailed sampling methodology is described in Gundacker et al. (50).

Ethics approval for the TRANSMIT data collection in 2023 was granted by the Ethics Commission at the Faculty of Humanities and Social Sciences, Humboldt Universität zu Berlin (approval no. HU-KSBF-EK_2023_0009).

### 2.2 Measurements

#### 2.2.1 Mental burden

**Depressive symptoms:** To assess the mental burden of respondents, two measurements were implemented in the survey. The Patient Health Questionnaire (PHQ) was used as a measurement for depressive symptoms. The PHQ is an internationally established self-assessment screening instrument for depression comprising nine items that assess the frequency of discrete symptoms related to sleep, appetite, and energy as defined in the DSM (see Table 1 for the items). In this study, we omitted the ninth item on suicidal thoughts. This practice is common in survey settings, where adequate psychological support cannot be provided if needed (51). The PHQ is available in 93 validated language versions on its official website, making it particularly popular in cross-cultural research. Although developers in the 1980s claimed the PHQ's adequacy for diagnosing major depression, it is currently used primarily as *the* screening and treatment monitoring tool in primary care and research settings (73). It is widely acknowledged that the PHQ overestimates depression (74), leading some scholars to argue that it should rather be interpreted as an indicator for depression severity (75) or general emotional distress and physical malaise [(76); c.f. (77)]^2^.

**Table 1:** Items of the Patient Health Questionnaire (PHQ) and the Feeling Broken or Destroyed Scale (FBD).

Patient health questionnaire (PHQ)	Feeling broken or destroyed scale (FBD)
*Over the last 2 weeks, how often have you been bothered by any of the following problems:*	*With reference to the past 2 weeks, how often have you…*
•Little interest or pleasure in doing things •Feeling down, depressed, or hopeless •Trouble falling or staying asleep, or sleeping too much •Feeling tired or having little energy •Poor appetite or overeating •Feeling bad about yourself—or that you are a failure or have let yourself or your family down •Trouble concentrating on things, such as reading the newspaper or watching television •Moving or speaking so slowly that other people could have noticed. Or the opposite—being so fidgety or restless that you have been moving around a lot more than usual.	•… felt that your spirit or morale is broken or destroyed? •… felt that your ambitions and hopes for the future are destroyed? •… felt emotionally or psychologically exhausted?

Each item was rated on a 4-point Likert scale, reflecting the frequency of described depression symptoms within the past 2 weeks (0 = not at all; 3 = nearly every day). Furthermore, the official English version of the PHQ was translated into Arabic by the professional research institute. A back-translation procedure was conducted to ensure consistency with the original English version and confirm translation precision. The items closely aligned with existing validated Arabic versions of the PHQ.

**Social suffering:** As a locally informed, Arabic-language measurement of social suffering, the Feeling Broken or Destroyed scale (FBD) was included as developed by Barber et al. (33). The scale comprises three items (see Table 1). Responses were provided on a 4-point Likert scale, indicating the frequency of suffering within the past 2 weeks (0 = not at all; 3= nearly every day), equivalent to the way the PHQ items were measured. While deviating from the original, this adaptation followed Barber et al.'s recommendation. Though developed among Palestinians, according to Barber et al., the FBD has demonstrated good psychometric properties among a sample of Egyptian youth, who also face protracted political and economic oppression. This study appears to be the first application of the FBD in a large-N survey among a displaced population and within Lebanon.

For both measures, the mean across all item responses was computed, with these values serving as primary outcomes of interest (mean PHQ and mean FBD). The analytical sample included only respondents who completed at least five PHQ items and all three FBD items.

#### 2.2.2 Socio-political context

Several questions addressed *socio-political context*: perceptions of risk of conflict in one's current area of residence, hopefulness for Lebanon's future, hopefulness for Syria's future, and experiences of racial/ethnic discrimination. For conflict risk perception, a binary indicator was created where respondent were classified as perceiving risk if they at least slightly agreed with the statement “I think there is a tangible threat of an armed conflict breaking out or extending to my current area of residence.” For hopefulness regarding Lebanon's and Syria's futures respectively, variables with three categories were constructed: yes, no, or “don't know.” Respondents who at least slightly agreed with the statement, “All things considered, I am hopeful that [Lebanon/Syria] is on a path for a better future,” were assigned a value of 1, while those who at least slightly disagreed received a value of 0. Respondents answering “don't know” constituted a separate third category. A binary indicator was also created for experiences of racial/ethnic discrimination, with 1 indicating reports of discriminatory threats or attacks due to racial and ethnic background occurring sometimes, fairly often, or very often, and 0 indicating never or almost never experiencing such incidents. Finally, based on the geolocation of PSU, the starting points for random walks within neighborhoods, and documentation of Israeli attacks on Lebanon from the Lebanese National Council for Scientific Research (CNRS) visualized by Public Works Studio (52), a variable was computed for each PSU indicating whether the neighborhood was located in a region exposed to direct threat of attacks at the time of data collection.

#### 2.2.3 Demographic and socio-economic indicators

Demographic indicators included age, gender, marital status, family separation, education level, and employment status. Family separation was defined as reporting that a spouse or a child under 18 is residing outside the household (including within the same city/province, another city/province, or another country), resulting in a binary variable.

In addition to these individual-level socioeconomic indicators, two further indicators of household and neighborhood conditions that capture the social context of economic deprivation were incorporated. Household economic status was derived from a question regarding the household's financial situation. Respondents were categorized as experiencing household poverty if they indicated that their household income was insufficient to afford food and/or other basic necessities. A binary indicator was created for neighborhood dwelling conditions, where respondents were classified as reporting poor neighborhood conditions if they slightly, moderately, or strongly disagreed that dwellings in their neighborhood were well maintained.

### 2.3 Analysis

Reliability measures and descriptive statistics were calculated for both mental burden measures. For the PHQ, the sum score of the eight items was first computed to identify respondents meeting clinical criteria for moderate or severe depressive symptoms (> or = 10) (51). Subsequently, mean PHQ and FBD scores were calculated. Bivariate correlations between PHQ and FBD were conducted to assess discriminant validity between the measures. Two linear regression models were then specified, with mean PHQ and FBD scores serving as the outcomes respectively. Covariates incorporated demographic, socioeconomic, and socio-political factors described previously. Hope variables for Syria and Lebanon were deliberately excluded from the regression analyses due to conceptual, potentially tautological overlaps with the outcome measures (see Table 1 for PHQ and FBD items). Instead, one-way ANOVA tests were employed: first, to examine significant differences in PHQ and FBD scores across the three categories (hopeful, not hopeful, ambivalent); seconds, to compare mean PHQ and FBD scores within each subgroup. All statistical analyses were performed using *R*.

## 3 Results

### 3.1 Sample

Our sample draws from respondents who indicated Syria as their country of origin in the 2023 TRANSMIT survey in Lebanon (initial *N* = 1,241). Due to October 7, 2023 marking a historical caesura with expected influence on the phenomena of interest in our study (see), we exclude 6 respondents who answered the survey between October 5 and 7. Finally, we only include respondents who answered all three FBD questions and at least five out of eight PHQ questions.

Descriptive statistics of the resulting final, cross-sectional analytical sample (*N* = 1,228) are presented in Table 2. Respondents have been living in Lebanon for an average of 10.0 years and range in age from 15 and 93 years old (mean = 34.8 years). The socioeconomic profile reflects conditions of acute precarity:

**Table 2:** Descriptive statistics on demographic, socioeconomic and socio-political characteristics.

Characteristic	*N* (%) or Mean (SD) (*N* = 1,228)
Age	
Mean (SD)	34.8(11.3)
Median [Min, Max]	33.0[15.0, 88.0]
Sex	
Male	609(49.6%)
Female	619(50.4%)
Marital status	
Single	238(19.4%)
Married	987(80.4%)
Missing	3(0.2%)
Educational attainment	
Never attended	177(14.4%)
Some school	578(47.1%)
Middle school cert.	303(24.7%)
High school cert.	109(8.9%)
Some university	21(1.7%)
Bachelor’s or higher	39(3.2%)
Missing	1(0.1%)
Family separation	
No	1,184(96.4%)
Yes	44(3.6%)
Employment status	
Unemployed	627(51.1%)
Employed	120(9.8%)
Day laborer	481(39.2%)
Household poverty	
No	121(9.9%)
Yes	1,106(90.1%)
Missing	1(0.1%)
Neighborhood not in good condition	
No	590(48.0%)
Yes	633(51.5%)
Missing	5(0.4%)
Risk of conflict	
No	600(48.9%)
Yes	620(50.5%)
Missing	8(0.7%)
Hopeful that Lebanon is on path for a better future	
Yes	461(37.5%)
No	498(40.6%)
Don’t know	246(20.0%)
Missing	23(1.9%)
Hopeful that Syria is on path for a better future	
Yes	451 (36.7%)
No	565 (46.0%)
Don't know	189 (15.4%)
*Missing*	23 (1.9%)
Discrimination experience	
No	797 (64.9%)
Yes	428 (34.9%)
*Missing*	3 (0.2%)
Years since arrival	
Mean (SD)	9.97 (3.97)
Median [Min, Max]	10.0 [0, 66.0]
*Missing*	35 (2.9%)
Residence in border region	
No	1,042 (84.9%)
Yes	186 (15.1%)
PHQ score	
Mean (SD)	1.17 (0.704)
Median [Min, Max]	1.00 [0, 3.00]
FBD score	
Mean (SD)	1.38 (0.784)
Median [Min, Max]	1.00 [0, 3.00]

Where missings are not listed, there are no missing values for that variable.

over 90% of respondents report experiencing household poverty, the majority were unemployed or working as day laborers, and around half report experiencing poor neighborhood conditions.

**Figure 1:**
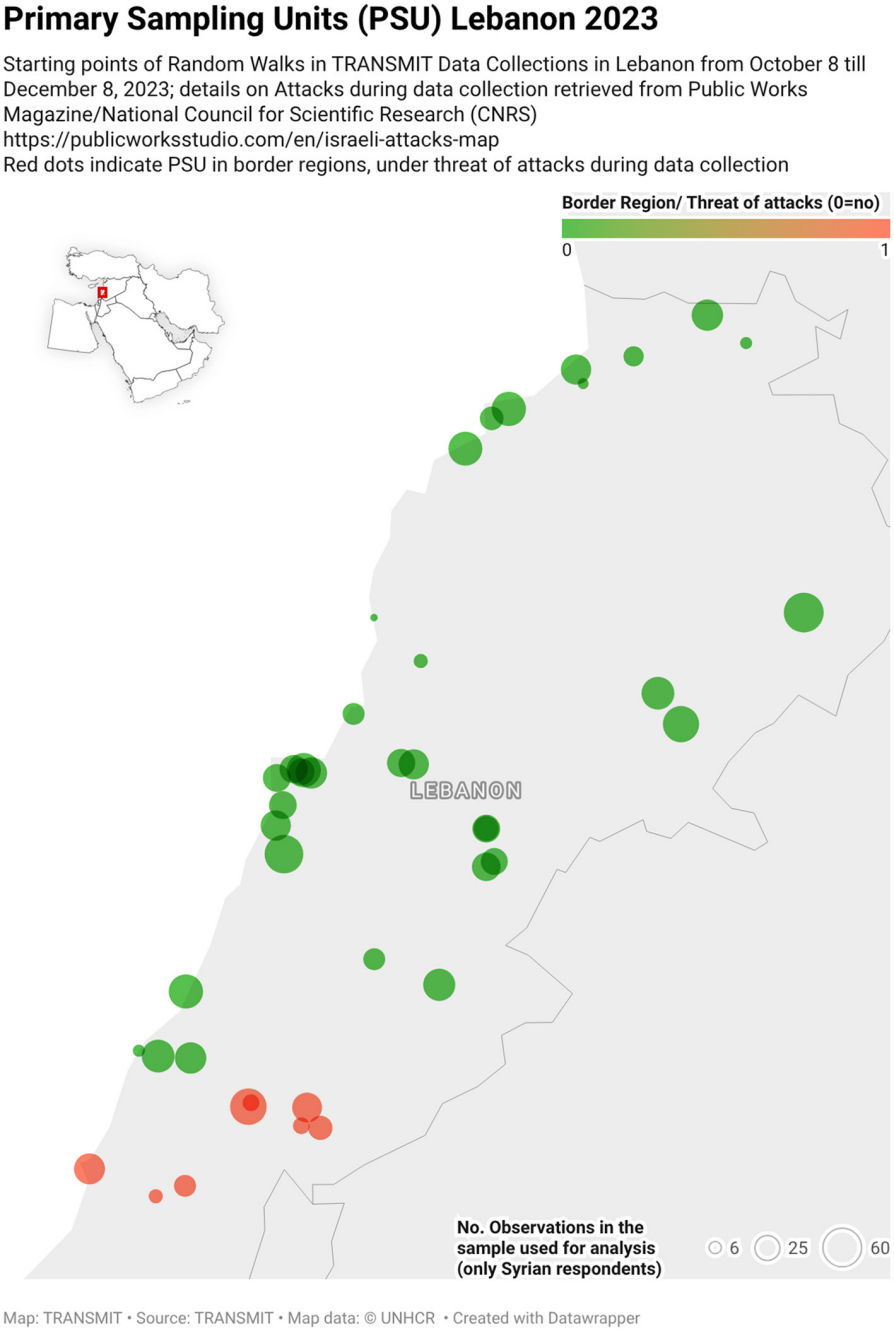
Geolocations of Primary Sampling Units. Details on attacks are retrieved from Public Works Magazine/National Council for Scientific Research (CNRS) https://publicworksstudio.com/en/israeli-attacks-map (accessed January 28, 2026). Red dots indicate those PSU in the area of active armed conflict at the time of data collection categorized as “border region”. For more details, see the online scalable version of the map (https://datawrapper.dwcdn.net/oFMJL/3/).

Regarding the context of social and political injustice, approximately one thid (34.9%) report experiences of discrimination based on race/ethnicity, and around half perceive a risk of armed conflict breaking out or extending into their current area of residence. Around one third of respondents expressed hopefulness about the futures of both Lebanon and Syria, and about 15% of the total sample (*N* = 186) respondents were residing in the southern regions exposed to immediate physical threats by armed conflict between Israel and Hezbollah. For more details on respondents' geolocations, see Figure 1 as well as the online scalable version of the map (https://datawrapper.dwcdn.net/oFMJL/3/).

### 3.2 Mental burden

**Figure 2:**
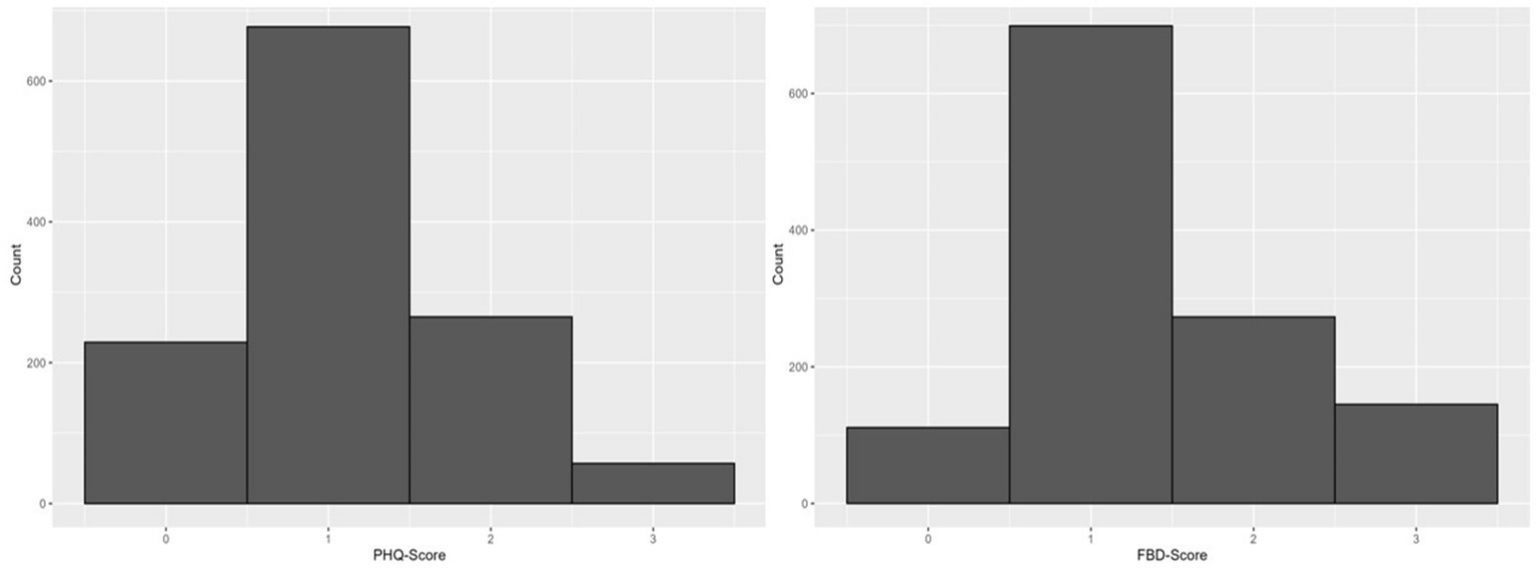
Distribution of mean PHQ and FBD scores.

Distribution of PHQ and FBD means scores are displayed in Figure 2. Both PHQ and FBD mean scores fell within the lower-to-mid range of their respective scales. Respondents reported higher levels of social suffering (FBD: *M* = 1.38, *SD* = 0.02, range = 0.00–3.00) than depressive symptoms (PHQ: *M* = 1.17, *SD* = 0.02, range = 0.00–3.00), a difference of 0.21 units that was statistically significant [*p* < 0.001, 95% CI (−0.251, −0.172), paired samples *t*-test]. When considering the sum score rather than the mean, 41.7% of respondents indicate a PHQ at or above the typical cutoff point of 10, indicating moderate to severe depressive symptoms. Though used for the first time within the Lebanese context and among a displaced sample, the FBD showed good internal consistency (Cronbach's Alpha = 0.822; Omega = 0.838) comparable to the results of Barber et al. (33). The PHQ showed good reliability with Cronbach's Alpha = 0.865 and Omega = 0.866. In addition, PHQ and FBD demonstrated moderate positive correlations in our sample (ρ =0.56, all *p* < 0.001), indicating that while the two scales are related—as expected for measures of mental burdens—they are sufficiently distinct to justify their use as separate constructs. These findings support the discriminant validity of the FBD scale.

### 3.3 Predictors of mental burden

Results of our regression analyses, displayed in Table 3, indicate that the FBD scale, assessing social suffering, may be more sensitive to some socio-political factors than the PHQ, assessing depressive symptoms. Most covariates indicating respondents' demographic background—including gender, age, and marital status—appear to have similar relationships with PHQ and FBD in terms of magnitude and significance. However, the relationship between family separation and FBD is significant (0.232, *p* < 0.05), while the relationship between family separation and PHQ lacks significance. While educational attainment appears not to be significantly associated with changes in PHQ and FBD, employment is associated with both decreased PHQ and decreased FBD compared to unemployment. Working as a day laborer, as compared to unemployment, is associated with increased PHQ, but not FBD.

**Table 3:** Results of OLS regression outputs with (1) PHQ and (2) FBD as dependent variables respectively.

Predictor	(1)	(2)
	PHQ	FBD
Intercept	0.544^***^ (0.118)	0.443^***^ (0.134)
Age	0.006^***^ (0.002)	0.005^*^ (0.002)
Female (ref. = male)	0.142^**^ (0.046)	0.199^***^ (0.053)
Married (ref. = single)	0.017 (0.045)	0.006 (0.052)
Family separation	0.041 (0.096)	0.232^*^ (0.110)
Years in Lebanon	−0.002 (0.005)	−0.009 (0.005)
*Educational attainment (ref. = never attended)*		
Some school	−0.041 (0.055)	0.083 (0.063)
Middle school certificate	−0.010 (0.060)	0.004 (0.069)
High school certificate	−0.001 (0.076)	−0.021 (0.087)
Some university	−0.006 (0.142)	−0.026 (0.162)
Bachelor's or higher	−0.074 (0.112)	0.022 (0.128)
*Employment status (ref. = unemployed)*		
Employed	−0.284^***^ (0.070)	−0.256^**^ (0.080)
Day laborer	0.165^***^ (0.048)	0.105 (0.055)
***Socio-political factors***		
Household poverty	0.098 (0.061)	0.330^***^ (0.070)
Neighborhood not in good condition	−0.010 (0.036)	0.146^***^ (0.041)
Risk of conflict	0.245^***^ (0.040)	0.301^***^ (0.046)
Discrimination	0.044 (0.038)	0.262^***^ (0.043)
Residence in border region	0.669^***^ (0.055)	0.469^***^ (0.063)
Observations (N)	1,174	1,174
*R* ^2^	0.280	0.243
Adjusted *R*^2^	0.269	0.232
AIC	2163.3	2473.2
BIC	2259.6	2569.5
Log-likelihood	−1062.7	−1217.6
F-statistic	26.448	21.873
RMSE	0.60	0.68

Unstandardized coefficients; standard errors in parentheses. ^*^p < 0.05; ^**^p < 0.01; ^***^p < 0.001.

Turning to socioeconomic and socio-political context, results indicate a significant relationship between FBD and household poverty (0.330, *p* < 0.001), poor neighborhood conditions (0.146, *p* < 0.001), and discrimination (0.262, *p* < 0.001). In our results, these covariates did not demonstrate a statistically significant link to higher PHQ. Two covariates—risk of conflict and residence in the border region—demonstrated statistically significant relationships with both higher PHQ and higher FBD. The magnitude of the association between residence in the border region and PHQ is approximately 1.5 times greater than the association between residence in the border region and FBD.

### 3.4 Hope for the Future

**Figure 3:**
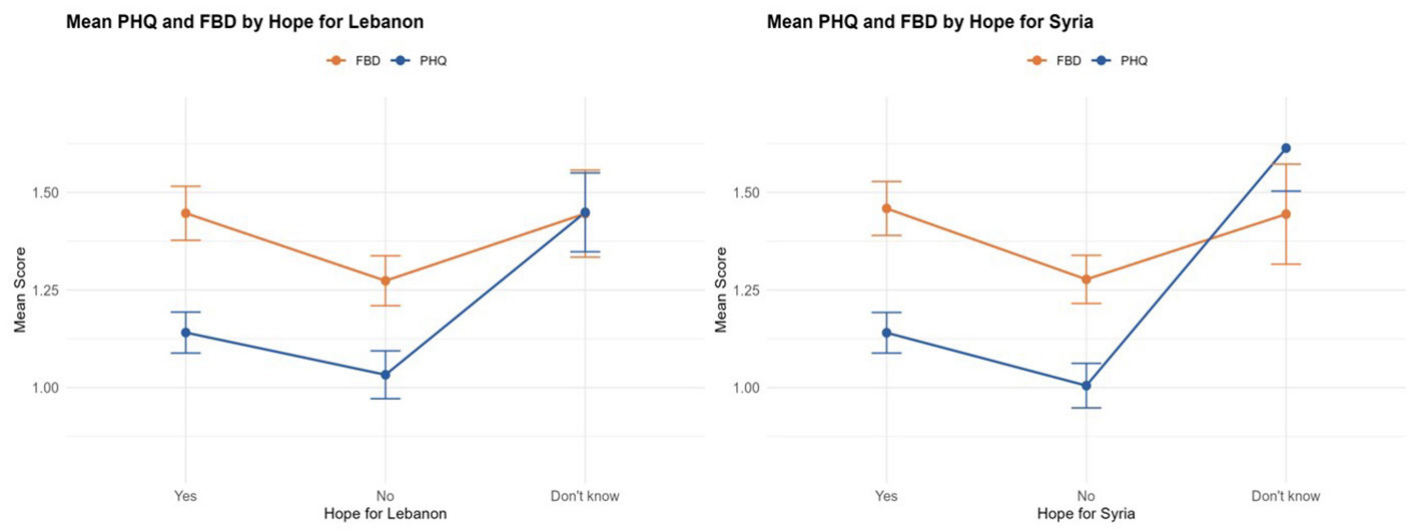
Mean PHQ and FBD by respondents' hope for Lebanon and Syria being on a path toward towardsa better future.

Regarding group differences based on respondents' attitudes toward Lebanon's and Syria's future, the pattern is more complex (see Figure 3). The highest levels of mean depressive symptoms (PHQ) are reported by respondents who are ambivalent about Lebanon's future (1.50), with significantly lower levels among both hopeful (1.14) and hopeless (1.03) respondents. ANOVA and *post-hoc* Tukey HSD test results indicate that the mean PHQ among respondents who answer “don't know” is significantly higher than both those who answer “yes” and those who answer “no,” The difference in mean PHQ between respondents who answer “yes” and those who answer “no” is not significant. Regarding FBD, assessing social suffering, patterns slightly differ: those who answer “yes” and those who answer “don't know” appear to demonstrate similar mean FBD levels (1.45 for both), while those who answer “no” have a mean FBD of 1.27. Mean FBD among respondents who answer “no” is significantly lower, indicating decreased social suffering.

Interestingly, quite similar patterns appear with regard to respondents' perceptions of hope for Syria: respondents who demonstrate ambivalence about Syria's future by answering “don't know” display a significantly higher mean PHQ (1.61) compared to those who indicate hope (1.14) or lack of hope (1.01), according to ANOVA and *post-hoc* Tukey HSD tests. When it comes to FBD, however, there are only significant differences between “no” and “yes” responses, with a mean of 1.27 and 1.46 respectively. The mean FBD of respondents who answer “don't know” (1.44) is not significantly different from the means of the other responses.

We finally compare PHQ and FBD to each other within response categories using paired *t*-tests. Among both hopeful and hopeless respondents, social suffering (FBD) is assessed at higher levels than depressive symptoms. However, these differences disappear when we compare PHQ and FBD among respondents who answered “don't know” to the question concerning hope for Lebanon's future. The difference between PHQ and FBD is statistically meaningful for respondents who answered “don't know” to the question concerning hope for Syria's future, with a mean PHQ of 0.17 points higher than mean FBD. We visualize the above-described results below in Figure 3.

## 4 Discussion

The explorative study presented examines the distribution and associated predictors of both social suffering and depressive symptoms among the displaced Syrian population that fled war in their country of origin and continues to live under conditions of chronic conflict, social injustice, and political injustice in Lebanon. We contend that the dominant focus on mental illness risks detaching mental burdens from the social world in which they are situated and might conceal and potentially render invisible the social suffering evident particularly among marginalized populations and “political catastrophes” (26). Our results serve as evidence of the FBD as a psychometrically sound instrument for evaluating social suffering in the displaced Syrian population in Lebanon. More importantly, our analyses indicate that social suffering, as operationalized by the FBD, represents a phenomenon distinct from depressive symptoms measured by the PHQ. This finding supports our theoretical framework and aligns with Barber et al.'s conclusions. It thereby highlights an imperative scholarly inquiry that moves beyond standardized symptom inventories to address mental burden in context.

The mental burden among Syrians is substantial, evidenced by 41.7% with moderate or severe depression based on the clinical interpretation of the PHQ. This finding aligns with—but methodologically differs from—Ruhnke et al. (9), who reported 26% with elevated mental burden in earlier TRANSMIT data (2020/2021). Longitudinal comparison (*n* = 60% re-interviewed in 2023 from prior surveys) suggests an increase in depressive symptoms among displaced Syrians in Lebanon, from 26% to 42%. Admittedly, Ruhnke et al., set the cut-off at PHQ >= 11, potentially underestimating the share of those with an elevated mental burden. As this represents the first published study utilizing the FBD since Barber et al. (33), we cannot compare the level of social suffering of displaced Syrians in our study to other samples from the same context. Future research should, equivalent to the emic development of the FBD, employ mixed-method approaches to explore displacement-specific and transnational aspects of social suffering and approach their structured assessment.

The results of our regression analyses indicate that older age, female gender, risk of conflict and residing in areas of active armed conflict are strongly associated with higher mental burden. In contrast, employment may function as a protective factor. However, this association requires further qualification: Working as a day laborer, typically characterized by the absence of formal contracts and concentration in the informal sector, is associated with increased depressive symptoms but shows no significant relation to social suffering. The majority of Syrian refugee workers in Lebanon are engaged in informal jobs, particularly in agriculture and construction, which are fields characterized by long working hours, low pay and complete lack of labor protections and irregularity (78). The divergence in the results between social suffering (FBD) and the depressive symptoms (PHQ) may suggest that the instability and the harsh working conditions characteristic of day laborer may trigger more immediate, individual-level manifestations of mental burden, such as fatigue, low mood and sleep disruptions. By contrast, social suffering appears less sensitive to specific employment conditions. Since they retain at least a minimal degree of financial security, their situation—though difficult—may lack the existential urgency characteristic of unemployment.

The findings regarding border regions require careful interpretation. In contexts of active armed conflict, the immediacy of threat to physical safety and survival may foreground acute, somatic and discrete symptoms, such as sleep disturbance or appetite changes, captured by PHQ. By contrast, social suffering, as assessed by FBD, reflects more enduring forms of mental burden, including apathy and exhaustion. These affective states might be temporarily disrupted or even overshadowed (as shown by the higher coefficient for PHQ than FBD) by the volatility and acute escalation of violence at the time of data collection.

Our interpretation is reinforced by the broader pattern of results: poverty, poor neighborhood quality, and experiences of discrimination demonstrate a statistically significant relationship with social suffering, but not with depressive symptoms. Although we cannot conclude that the structural conditions captured in our cross-sectional analyses causally contribute to increased social suffering, we can interpret the differences in correlations described above as further empirical support for treating the two constructs as conceptually distinct. While PHQ may be more responsive to acute and immediate stressors, social suffering may more directly capture the effects of structural vulnerabilization (79, 80), insofar as it reflects the cumulative impact of material deprivation, social exclusion, and marginalization. The concept of *structural vulnerabilization* reminds us that vulnerability is not a passive state of risk, but a dynamic produced by socio-economic hierarchies, political marginalization, and systemic inequities (79, 80). *Social suffering* becomes hence a lens for understanding how structural forces are internalized and embodied, producing forms of suffering that are relational and rooted in unequal power relations. That the FBD, and not the PHQ, appears to capture this dimension empirically lends methodological weight to the argument that standard psychiatric screening instruments, however useful for identifying clinical need, are in some cases not equipped to render visible the social and political conditions that produce suffering in the first place.

Moving beyond our empirical evidence, we discuss how social suffering allows researchers to better understand the relational intersubjectivity of suffering, for instance, by acknowledging its potential to create or “ruin the collective and intersubjective connections of experience” (26, p. x). The relational dimension becomes particularly visible in how distress is expressed in specific socio-political contexts. For example, studies conducted in Palestine show that suffering is often expressed through pluralized cultural idioms as *Makhnogeen* (feeling suffocated), *Masjoneen* (feeling imprisoned), and *Maazoleen* (being segregated), which convey socially embedded and collectively lived conditions under blockade and violence (53).

Building on this relational understanding of suffering, Lamia Moghnieh, whose research as a mental health practitioner and medical anthropologist centers on Lebanon, pushes the analysis further by demonstrating how social suffering is also differentially recognized or silenced through its interrelational reproduction within social, moral, and institutional contexts. Her fieldwork in Southern Lebanon demonstrates how the international humanitarian psychiatry community's assessment of trauma absence in Lebanese society following the July 2006 war was erroneously equated with an absence of suffering. This equation concealed the profound wounds and harmful experiences stemming from exposure to a war that claimed over 1,100 lives (predominantly civilians) and displaced nearly one million individuals. It was only several years later, through the presence of displaced Syrians, that Lebanese started openly expressing the traumatic quality of their suffering. Moghnieh explains the inter-relational (re)production of suffering as follows:

In the case of the Syrian refugee crisis, however, humanitarian agencies such as the United Nations Higher Refugee Council (UNHCR) now engaged in bureaucratic work to identify evidence of true victimhood. PTSD constituted such evidence, becoming a clear indicator through which Syrians were recognized as legitimate victims. Psychiatrists were asked to prepare what Erica James refers to as “trauma portfolios” –the aggregate of documentation and verification which “recognizes” or transubstantiates individuals, families or collective sufferers into “victims” and “survivors”' [(81), p. 131]. A PTSD diagnosis was central to this portfolio and became a clear ticket allowing access to aid services. […] This new political economy of trauma radically shifted the politics of suffering in Lebanon. Within this context, Lebanese communities now struggled to have their own suffering recognized as traumatic. This was evident in the daily encounters I observed between Syrians and Lebanese – in public taxis and cafés, at the supermarket and the post office, in the waiting rooms of clinics – where Lebanese people competed to show how their own suffering was greater than the Syrians'. They would angrily recite past stories of violence, especially from the Lebanese Civil War, now expressing a suffering not previously shared in public. […] Such daily recollections of violence repeatedly de-legitimized the suffering of Syrians. These recollections were intimately tied to competing economies of trauma and aid in Lebanon. The presence of Syrians thus prompted the sharing of past experiences of violence that had rarely been narrated in the public sphere or framed as individual injuries and claims. (14, p. 18)

What appears as a “competitive” narrative of suffering is structurally produced by the political economy of humanitarian aid, in which institutions such as the UNHCR require standardized, legible forms of victimhood, most prominently the diagnosis of PTSD, as a prerequisite for accessing resources. This is precisely the dynamic Fassin documents in his work on Humanitarian Reason, where he shows how humanitarian governance privileges certain expressions of suffering over others, thereby producing hierarchies of deservingness (54). In this sense, the language of suffering becomes the primary currency through which access to protection is guaranteed, pointing to the differential distribution of humanitarian recognition.

The practices and partial imperative of the “vulnerability contest” (55), depicted by Moghnieh for the Lebanese context specifically, are globally evident. The fixation on refugees' vulnerability has been harshly criticized by many scholars and practitioners (12–14, 22, 56, 57). This critique, besides other aspects, questions the normative response and counter-concept to vulnerability, which is “humanitarians' aim to turn ‘vulnerable' refugees into ‘resilient' ones” (58, p. 6). Fostering resilience constitutes a fundamental component of numerous Mental Health and Psychosocial Support (MHPSS) schemes in post-emergency contexts and refugee camps. *Resilience* thereby describes the individual's or community's capacity to adapt, cope and make meaning of the crises, conflicts and wars of the past, and the present and prepare for those of the future (32, 56)—instead of scrutinizing, negotiating, and fighting the immense social and political injustice and violence they are exposed to.

Notably, our empirical findings reveal a counterintuitive pattern: respondents who report a complete absence of hope for the future of both Lebanon and Syria exhibit lower levels of mental burden compared to those who remain ambivalent or hopeful. At first glance, this finding appears to contradict dominant resilience frameworks, in which hope is broadly treated as a protective factor against suffering and mental illnesses (32). Resilience-oriented interventions accordingly position the promotion of hope as a core component (58). Our empirical findings, however, point to a more ambiguous relationship. The loss of hope may, in some cases, function as a form of acceptance or affective disengagement—a state preceded by profound mental exhaustion that paradoxically corresponds with reduced mental burden. Maintaining hope or occupying a position of ambivalence may, by contrast, demand the continuous labor of imagining alternative futures and sustaining a sense of responsibility toward them. Hope, in this sense, is not simply protective; it also renders individuals more susceptible to disappointment, frustration and emotional strain, which may manifest in depressive symptoms such as insomnia, feelings of guilt or worthlessness.

These findings resonate with a growing body of scholarship across sociology, philosophy and political theory that underscores the complex and ambivalent nature of hope (59–61). Such ambivalence may be most precisely captured by the formulation of hope as “fearing the worst but yearning for better” [(62), p. 282]—a definition that foregrounds the tension, rather than the optimism at hope's core. Drawing on this reasoning, we suggest that hope can, under certain conditions, become a site of tension between possibility and constraint—a tension that those who have relinquished hope no longer bear.

This understanding draws attention to the simultaneous anticipation and vulnerability inherent in hope as an affect, which dominant discourse too often reduces to a proxy of positivity and optimism. Just as an exclusive focus on mental ill-health risks obscuring forms of social suffering, resilience frameworks risk flattening the complexity of hope. Resilience interventions may reproduce a logic of adaptation that erodes the longing for a genuinely different future (82). What emerges in contexts where resistant or even utopian aspirations persist under conditions of sustained violence and socio-political injustice, we argue, cannot be adequately captured through the lens of resilience. Yet debates on mental suffering are often quickly channeled into resilience frameworks, thereby limiting the conceptual space for alternative ways of understanding how people endure and respond to injustice. In contrast to the normative pairing of vulnerability and resilience, we therefore propose resistance as a complementary analytical lens that opens up rather than constrains analytical and political imaginaries. Resistance can coexist with, or even be articulated through experiences of emotional strain and vulnerabilisation, without being reducible to the absence of mental burden.

The question then shifts: rather than asking how individuals cope with suffering, or adapt to the structural vulnerabilisation they face (63), we ask how “trajectories of suffering” and the socio-political conditions in and from which suffering emerges are recognized, named, resisted and changed. This framing centers the active subject rather than the vulnerable object, and acknowledges the embedded, relational nature of both suffering and response. Crucially, resistance does not need to take the form of collective action alone; it may equally be an inner state of steadfastness on the individual or the collective level [c.f. *sumud* among Palestinians and Lebanese in response to Israeli violence and occupation (14, 64)]. Embedded qualitative research is required to illuminate the individual and collective practices through which hope and resistance are lived, and to examine their relationship to mental burden, particularly among displaced populations and in contexts of chronic violence and injustice.

### 4.1 Limitations

There are several limitations to the study that should be mentioned. While the FBD scale quantifies individual experiences of social suffering, its structured format may not fully encompass the collective framing (“we”) commonly used to narrate such suffering in natural discourse (53). This methodological gap suggests that the potential of novel measurement approaches to adequately represent this collective dimension requires further investigation, alongside alternative methodological approaches that gather data on a more collective basis. Likewise, the illumination of intersubjectivity, the way in which suffering is recognized, denied or produced through relations between individuals and communities, requires in-depth qualitative methodologies. At the same time, structured quantitative approaches can complement this inquiry. One possible strategy is the structured assessment of social suffering in the Lebanese society using the FBD scale, and the parallel analysis of its predictors. This can offer genuine insight into this relational aspect as well as the distribution of suffering across both populations within the same socio-political context.

In addition, statistical limitations to the analysis should be taken into account. Most significantly, while the PHQ scale is composed of eight items, the FBD scale is composed of just three items. This limits statistical comparability between the two scales, as longer scales tend to have higher internal consistency (i.e., Cronbach's alpha), simply due to the number of items. The high internal consistency demonstrated by FBD may partly reflect item proximity rather than construct coverage. We consequently suggest that future research may adopt more robust approaches to comparing the two scales by accounting for the length disparity. In addition, we propose that further development of a measure that includes more items and reflects the many political and social conditions that shape experiences of social suffering would be a worthy scholarly venture. Here, we study whether a three-item scale can appropriately reflect several distinct factors related to socio-political context. A more refined and detailed measure of social suffering that includes more items may better capture the nuances or specificities of these different factors.

Finally, as only a small proportion of the sample indicated experiencing family separation (3.6%), respective results should be interpreted with caution.

### 4.2 Outlook: continuity of violence and displacement in the Lebanese context

The findings presented here are situated within an ongoing continuity of violence and displacement in the Lebanese context, in which social suffering and depressive symptoms were examined using data from fall 2023. By fall 2024, Israeli military operations expanded toward the center of Beirut, and had displaced an estimated 1.3 million people, with 620,000 crossing into Syria—then still under the Assad regime—before the November 2024 ceasefire (65, 66). Notably, 37% of these crossings were Lebanese nationals, illustrating what Fiddian-Qasmiyeh (22, 67) terms “overlapping refugeedom”: displacement, both internal and international, as repetitive and continuous rather than singular or bounded.

Following Hezbollah's response to Khamenei's assassination by launching attacks on Israel on March 2, 2026, and its involvement in the U.S. and Israel's war on Iran, Lebanon is once again experiencing a renewed peak of large-scale violence. Israel intensified aerial bombardment and launched a ground invasion in the southern border regions aimed at establishing a “security zone” extending to the Litani River, affecting almost one-tenth of the Lebanese territory, an area even larger than the buffer zone under Israeli occupation in 1978 (68). With this in mind, UN Secretary-General António Guterres urged that “Gaza model must not be replicated in Lebanon” (69). By March 2026, nearly 20% of Lebanon's population, was displaced (70). Syrian refugees, already among the most vulnerable, faced disproportionate impact (71, 72). Lebanon and the region more broadly thus remain affected by ongoing displacement, social and political injustice.

This continuity of violence, displacement and injustice in Lebanon and the broader region makes it particularly relevant to further conceptualize and study social suffering beyond individual psychopathology, just as hope and resistance beyond resilience. By broadening the analytical lens and highlighting the social suffering of the displaced Syrian population in Lebanon, we contribute to what Giacaman (19) calls the “simultaneous personalization of war and politicization of health”—suffering as inseparable from the socio-political conditions in and from which it emerges.

## Data Availability

The datasets presented in this study can be found in online repositories. The names of the repository/repositories and accession number(s) can be found below: DeZIM.fdz DataSearch [https://datasearch.fdz.dezim-institut.de/item/de.dezim/0179b282-f8b1-4bc9-accd-4fe9aecfc02e]. DeZIM.fdz DataSearch enables you to request access to the data of interest.

## Footnotes

1 Pierre Bourdieu's earlier work in *The Weight of the World: Social Suffering in Contemporary Society* established the concept of social suffering through empirically detailed case studies, demonstrating its analytical utility. Building upon this foundation, Emmanuel Renault subsequently develops a sophisticated typology of social suffering, advancing theoretical categorization beyond descriptive accounts. Renault defines social suffering as a transversal, dynamic and contextual “connect[ion of] social suffering in the sense of the subjective harm of ordinary socialization and social suffering in the sense of psychological disorders with a specifically social aetiology” (p. 31). Renault further differentiates the dynamics of social suffering from its content, specifically distinguishing between physical suffering (concerning the physical self), psychological/psychic suffering (concerning the psychological self) and psychosocial suffering (concerning the social self).

2 In Ruhnke et al. (9), presenting a study from the same project (TRANSMIT), we interpreted the PHQ as a measure of unspecified mental distress situated within the biomedical paradigm. For methodological clarity and explicit differentiation between the PHQ and FBD instruments, we adopt the interpretation of the PHQ as a measure of depressive symptoms in this manuscript.
